# Single-Crystal X-ray and Solid-State NMR Characterisation of AND-1184 and Its Hydrochloride Form

**DOI:** 10.3390/ma14237175

**Published:** 2021-11-25

**Authors:** Tomasz Pawlak, Małgorzata Szczesio, Marek J. Potrzebowski

**Affiliations:** 1 Centre of Molecular and Macromolecular Studies, Polish Academy of Sciences, Sienkiewicza 112, 90-363 Lodz, Poland; marekpot@cbmm.lodz.pl; 2 Institute of General and Ecological Chemistry, Faculty of Chemistry, Lodz University of Technology, Żeromskiego 116, 90-924 Lodz, Poland; malgorzata.szczesio@p.lodz.pl

**Keywords:** API, quantum-chemical calculations, GIPAW, CASTEP, very fast MAS NMR, molecular dynamics

## Abstract

In this study, we report on a structural investigation of AND-1184, with the chemical name N-[3-[4-(6-fluoro-1,2-benzoxazol-3-yl)piperidin-1-yl]propyl]-3-methylbenzenesulfonamide (**MBS**), and its hydrochloride form (**MBS**^HCl^); AND-1184 is a potential API for the treatment of dementia. The single-crystal X-ray investigation of both forms results in monoclinic crystal systems with P2_1_/c and C2/c symmetry for **MBS** and **MBS**^HCl^, respectively. This solid-state NMR study, combined with quantum-chemical calculations, allowed us to assign all ^13^C and most ^1^H signals. The **MBS** structure was defined as a completely rigid system without significant dynamic behaviours, whereas **MBS**^HCl^ exhibited limited dynamic motion of the aromatic part of the molecule.

## 1. Introduction

In-depth structural research on drugs and their active pharmaceutical ingredients (APIs) is required more today than in the past. In 2018, a record-breaking number of over 1000 drugs were introduced to the market, over 60 of which were completely new proposals. This trend has become more and more visible from year to year [1]. Synthesis, as well as biological aspects of synthetised API, are most frequently reported, without structural studies of their solid-state compositions. However, it is well known that different forms of the same pharmaceutically active substance can also differ in their physical properties which may influence pharmaceutical applications [2,3]. Accordingly, the importance of knowledge regarding the differences in drugs’ physical forms has been growing [4,5,6].

From an experimental point of view, we can use several techniques to investigate the solid-state composition of materials. Solid-state magic-angle spinning (MAS) NMR spectroscopy [7,8] is one such effective method for determining structure at the atomic level, short-range interactions, or the dynamics of solids. It probes the local atomic environments and does not require long-range order to have constructive conclusions [9]. This explains its wide application nowadays in many fields, from materials to pharmaceutical chemistry [8]. In the latter, the investigation of conformation, as well as crystal packing, is essential to understand the structure–property relationship that can have an influence on the formulation during drug development.

**Scheme 1:**
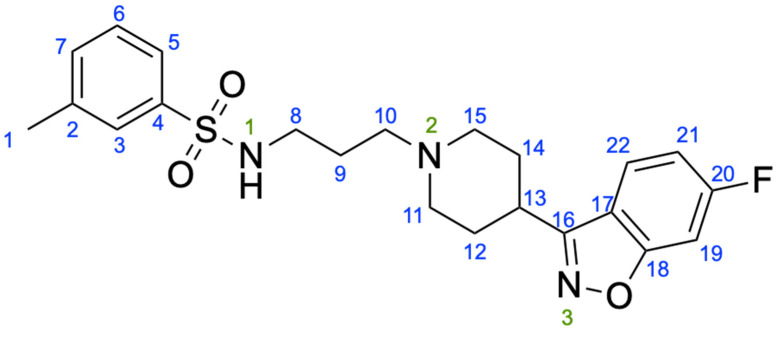
Chemical structure of **MBS** with numbering system.

In our study, we aimed to report on a solid-state study of N-[3-[4-(6-fluoro-1,2-benzoxazol-3-yl)piperidin-1-yl]propyl]-3-methylbenzenesulfonamide (**MBS**) and its hydrochloride form (**MBS**^HCl^) based on single-crystal X-ray, solid-state NMR, and quantum-chemical calculations. The chemical structure of **MBS** is shown in Scheme 1. This compound is named by code AND-1184 and is not a commercially marketed API; it is still under development and indicated for the treatment of the signs and symptoms of dementia [10,11]. From a chemical point of view, **MBS** is an indolamine derivative ligand receptor 5-HT_6/7_ antagonist [10]. To date, dementia is an incurable central nervous system disease, which is connected with many severe behavioural and psychological symptoms [11,12,13]. Antipsychotic drugs are frequently given, but most of them interfere with cognitive function, which is an additional problem for dementia-suffering patients [14]. Therefore, there is a strong need to develop APIs with antipsychotic activity, which do not have side effects that cause additional cognitive problems.

Although **MBS** and **MBS**^HCl^ are still waiting for approval for clinical studies, it is not unusual; the delay between discovery and medical application is counted in years for most new proposals. Until now, in the literature, there have been no solid-state studies of this API except the single-crystal X-ray structure of **MBS**^HCl^. The aim of this study is to perform a structural investigation and extend the knowledge about the MBS as well as **MBS**^HCl^, and therefore simplify their potential applications. 

## 2. Materials and Methods

### 2.1. Synthesis and Crystallisation of **MBS**

The **MBS** investigated compound was synthesised by JINAN YSPharma Biotechnology Co., Ltd., Company. The purity of **MBS** was >98%, which was confirmed by HPLC and solution NMR measurements. The crystalline **MBS** sample was obtained by crystallisation in the chloroform solution at 5 °C. The **MBS**^HCl^ crystals were obtained through the reaction of the **MBS** compound and HCl with a 1:1 molar ratio in ethanol at 50 °C and left through crystallisation. It was also possible to obtain the **MBS**^HCl^ through a mechanochemical reaction between HClaq and **MBS**.

### 2.2. Crystal Structure Determination

The single-crystal X-ray measurements were performed on a diffractometer (XtaLAB Synergy, Dualflex, Pilatus 300 K, Rigaku Corporation, Tokyo, Japan) at 100 K. Data reduction was conducted using the CrysAlisPro program (Agilent Technologies UK Ltd., Yarnton, UK) [15]. The SHELX was used for structure refinement [16,17]. Non-hydrogen atoms were anisotropically refined. The drawings and geometrical parameters were obtained using the programs Mercury [18] and PLATON [19].

### 2.3. NMR Spectroscopy

All solid-state magic-angle spinning (MAS) NMR experiments, cross-polarisation (CP) as well as one-pulse ^1^H or ^19^F, were performed on a 600 MHz Avance III spectrometer. The operating resonances were 600.13, 564.68, 150.90, and 60.811 MHz for ^1^H, ^19^F, ^13^C, and ^15^N, respectively. The experiments were conducted on HX MAS probe heads by using 2.5 and 4 mm ZrO_2_ rotors.

The Hartmann–Hahn conditions for ^13^C and ^15^N were set on a ^13^C, ^15^N-labeled histidine hydrochloride sample. The ^13^C CP MAS and ^13^C DD CP MAS spectra were performed with a CP contact time of 2 ms. For CP, a ^1^H ramp shape with a 90% to 100% setup and an RF of almost 63.5 kHz was used. A proton 90° pulse length of 4 μs, a spectral width of 40 kHz, a time-domain size of 3.5 k data points, and a repetition delay of 60 and 10 s were set up for **MBS** and **MBS**^HCl^, respectively. The ^13^C DD CP MAS was acquired with the τ_DD_ period of 50 μs. A SPINAL-64 decoupling sequence was used during acquisition time [20]. A proton 90° pulse length of 2.5 μs, a spectral width of 250 kHz, and a repetition delay of 45 s were set for the ^19^F one pulse MAS experiment.

The PISEMA MAS experiment [21,22,23] was conducted using a ^1^H pulse strength of 50 kHz. We adjusted the ^13^C spin-lock field strengths to the first-order sideband condition, ω_13C_ = ω_1H_ ± ω_r_. The spinning frequency was set to 13 kHz ± 3 Hz. We acquired 256 co-added transients for each of 64 *t*_1_ FIDs what corresponds to a 23 h experimental time. The 2D PISEMA MAS experiments incremented the SEMA contact time to a maximum *t*_1_ evolution time of 1 ms. The SEMA contact time was changed with a step of 16.28 μs. The data were transformed using the Bruker TopSpin 3.5 program software [24].

The fast MAS spectra were recorded on a 600 MHz Bruker Avance III spectrometer. The operating resonances were 600.13 and 150.90 MHz for ^1^H and ^13^C, respectively. The experiments were carried out on an HCN MAS probe head operating in double-resonance mode using 1.3 mm ZrO_2_ rotors. The spin rate was 60 kHz in all experiments. The pulse sequence for the ^13^C–^1^H invHETCOR (for indirect detection of ^13^C) experiments was described elsewhere [25,26,27]. The following parameters were used: 2 ms, 100 μs, and 2.5 μs, for a first contact time, a second contact time, and a proton 90° pulse length, respectively. Both ^1^H CP pulses were collected with a ^1^H ramp shape strength from 0.9 to 1.0. Both cross-polarisation pulses were set up to be 160 and 109 kHz for ^1^H and ^13^C, respectively. An SWF-TPPM decoupling sequence was used during acquisition time [28,29]. The ^1^H decoupling was set up to nutation frequency as low as 10 kHz and a pulse length of 50 μs [20]. The ^13^C–^1^H invHETCOR experiment was performed up to maximal evolution times of *t*_1max_ = 12 ms and *t*_2max_ = 10 ms. A total experimental time was 20 h what corresponds to 450 t_1_ FIDs for every 32 co-added transients. The sign discrimination was used by the States-TPPI method [30].

The ^13^C chemical-shift scale was referenced to Adamantane (resonances at 38.48 and 29.46 ppm) [31,32]. The powdered ^15^N glycine (resonance at δ = 34.40 ppm) was used as an external secondary reference to neat liquid ammonia [32,33]. The PTFE (resonance at δ = −122.7 ppm) was used as a secondary reference standard to CCl_3_F [32].

### 2.4. QM Calculations

The CASTEP 19.11 code was applied for all DFT calculations with periodic boundary conditions [34]. The energy convergence limit was set to 10^−7^ eV. In all cases, as the input files, the single-crystal X-ray diffraction structures were used. All the calculations were performed by applying the MBD* dispersion correction scheme (DFT-D method) and the PBE functional [35,36]. The maximum plane-wave cutoff energy was 630 eV with ultrasoft pseudopotential [37]. The optimisation algorithm was BFSG [38]. The Brillouin zone was sampled by the Monkhorst−Pack grid method [39]. The gauge including the projected augmented wave (GIPAW) method was used for the computation of all NMR chemical shifts [34,40,41]. Finally, we obtained NMR chemical-shielding values in periodic boundary conditions. NMR chemical-shielding values were recalculated to the chemicals shifts by applying the linear regression between calculated and experimental results. 

## 3. Results

### 3.1. Single-Crystal X-ray Determination of the **MBS** and Its Comparison with the **MBS**^HCl^ Form

**Figure 1:**
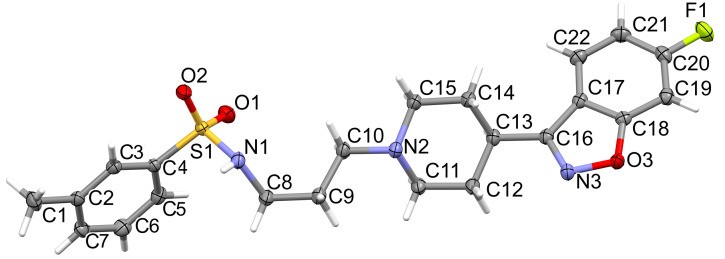
The molecular structure of **MBS** including the atom-labelling scheme. Displacement ellipsoids are drawn at the 50% probability level and H atoms are shown as small spheres of arbitrary radii.

The crystal data, details of data collection, and structure’s refinement parameters are summarised in Table 1. The structure is deposited in CCDC under no. 2115171. The molecular structure of the studied compound is shown in Figure 1. The compound crystallised in the monoclinic P2_1_/c space group. The crystal was in the form of a colourless plate. The asymmetric unit consists of one molecule.

**Figure 2:**
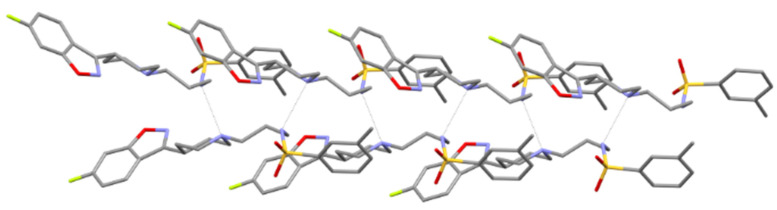
The intermolecular hydrogen bonds in **MBS**.

**Figure 3:**
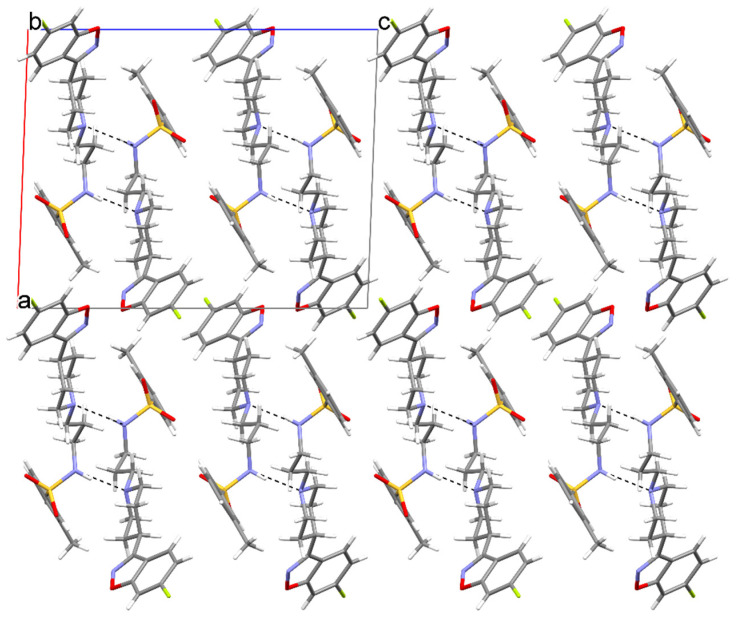
The crystal packing of **MBS**, viewed along the b axis, where (**a**–**c**) denote unit cell axes.

The N1–H1^...^N2 hydrogen bond connects molecules into the infinite chain C(6) according to graph-set analysis (Table 2 and Figure 2) [42]. Additionally, weaker hydrogen bonds of the C–H^...^O type are formed with the SO_2_ group (Figure 3).

**Figure 4:**
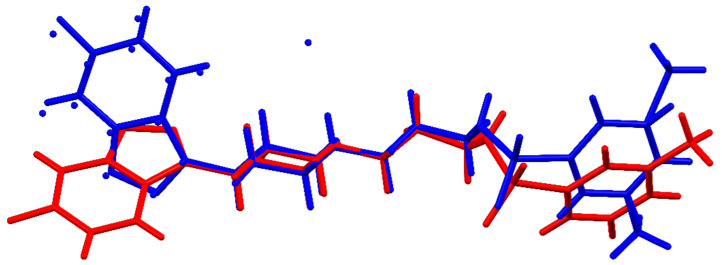
Overlay of **MBS** (red) and **MBS**^HCl^ (blue) molecules extracted from their crystal structures.

**Figure 5:**
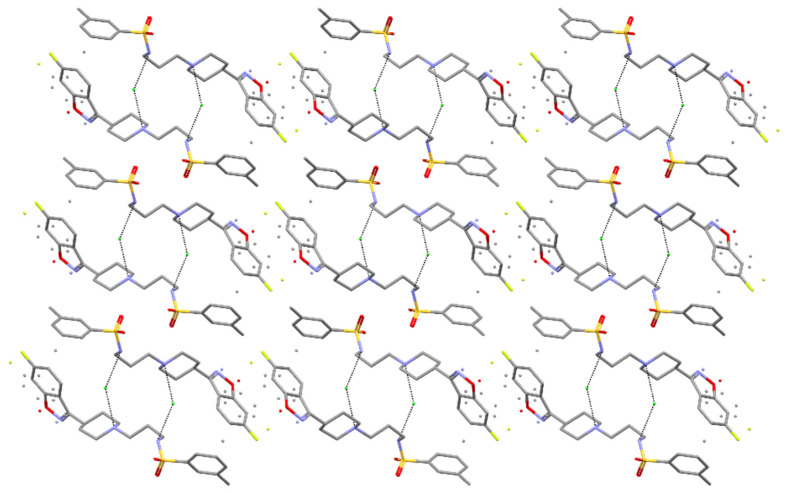
The crystal packing of **MBS**^HCl^, viewed along the *b* axis. Coordinates taken from [44].

In the crystallographic base of CSD [43], there is a compound in the form of hydrochloride (WINKIQ) [44], denoted in this study as **MBS**^HCl^. The protonation of the molecule occurs on nitrogen in pyridine. This compound is also crystallised with one non-dependent molecule in a monoclinic crystal system. In this structure, the molecule is disordered on the aromatic ring system. The disorder also occurs on the six-membered ring due to rotation around the C-S bond. Molecules assume a similar conformation, differing in the arrangement of the two-ring system (Figure 4). The chlorine atom creates two hydrogen bonds with NH groups (from pyridine and from the molecule chain), and a chain of hydrogen bonds is created (C1,2 (8)). In both structures, there is a chain of hydrogen bonds, which causes a similar packing of molecules in the direction of the *b* axis, and, in the case of a protonated compound, the chlorine atom creates a ‘bridge’ between the molecules (Figure 5).

### 3.2. Solid-State NMR and DFT-D Characterisation of the **MBS** and **MBS**^HCl^

**Figure 6:**
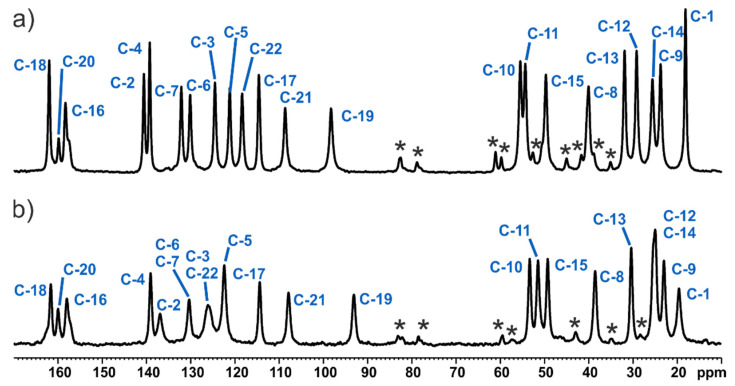
^13^C CP MAS NMR spectra of **MBS** (**a**) and **MBS**^HCl^ (**b**) recorded at a spinning rate of 8 kHz at an ambient temperature. Asterisks indicate spinning sidebands.

The solid-state NMR investigation of **MBS,** as well as the **MBS**^HCl^ forms, which were started from the analysis of the ^13^C CP MAS spectra, is shown in Figure 6. It can be observed that, for both structures, the number of resonance lines matches quite well the number of carbons in the molecule. The plots show significant differences, allowing us to quickly distinguish and identify both studied objects. The observed chemical distinctions between both forms are also reflected on the ^19^F MAS NMR spectra shown in the Supplementary Materials (Figure S1).

The same quality of **MBS**^HCl^ material was obtained through the classical reaction between **MBS** and HCl in ethanol, as well as in the mechanochemical synthesis, by using ball milling. The second method is especially interesting since it is reduced to the minimum number of necessary components by eliminating solvent from the reaction area, which is very desirable and reflects a green chemistry philosophy.

The high crystallinity of materials is apparent and appears slightly better for **MBS** considering the shape of peaks. The aromatic signals of **MBS**^HCl^ are broad and partially overlapped. These features, as well as much less intensity of the C-1 (-CH_3_ group) signal for the **MBS**^HCl^ than for the **MBS** sample, are understandable, taking into consideration the disorder appearing in this part of the molecule, which was discussed in the previous section by using the single-crystal X-ray method. Since molecular dynamic processes usually cause a decrease in the magnetisation transfer efficiency, we performed a molecular dynamic investigation by using the PISEMA MAS NMR sequence. Our observations show no evidence of a high amplitude molecular dynamic process of both studied objects. However, we cannot exclude small-angle wobbling (<180°) of the aromatic part of the molecule in **MBS**^HCl^. More details are included in the Supplementary Information.

**Figure 7:**
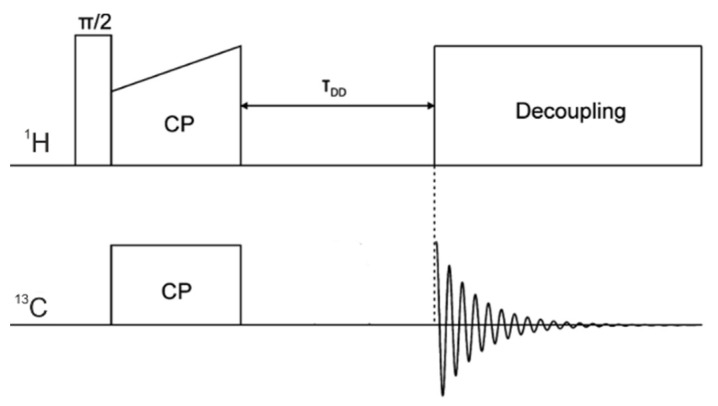
^13^C DD CP MAS NMR pulse sequence.

**Figure 8:**
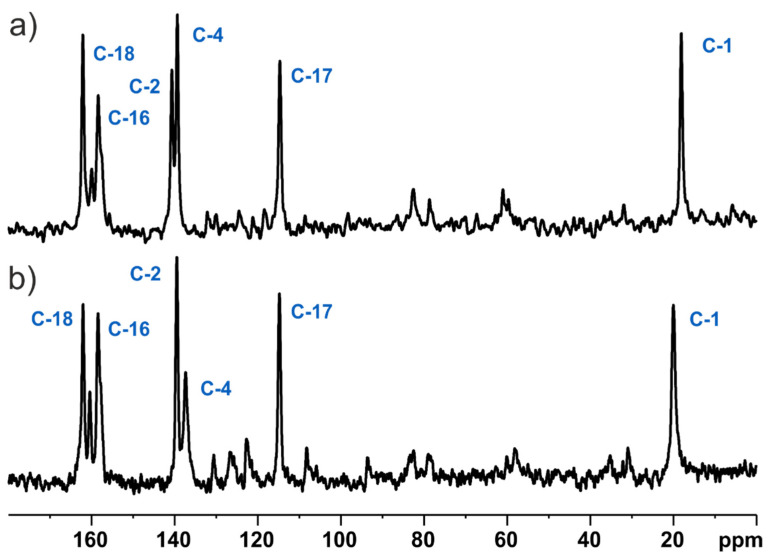
^13^C DD CP MAS NMR spectra of **MBS** (**a**) and **MBS**^HCl^ (**b**) recorded at a spinning rate of 8 kHz at an ambient temperature. Dephasing delay was 50 μs.

The presented assignment of signals (Figure 6) was supported by characterising the protonation state of carbons. Especially, it was important for a crowded part of spectra where it was not easy to predict the correct order of signals. For this purpose, we used a spectral-editing technique in the dipolar dephasing experiment [45]. The ^13^C DD CP MAS pulse sequences are shown in Figure 7. The spectra (Figure 8) were acquired without proton decoupling at the time of the τ_DD_ period. As a result, we obtained only the resonances with weak dipolar coupling to protons as methyl and quaternary carbons. Finally, there are no overlapped peaks between the ^13^C DD CP MAS and ^13^C CP MAS spectrum that belong to categories different from those previously mentioned (i.e., they are exclusively =CH- and -CH_2_- carbon signals).

**Figure 9:**
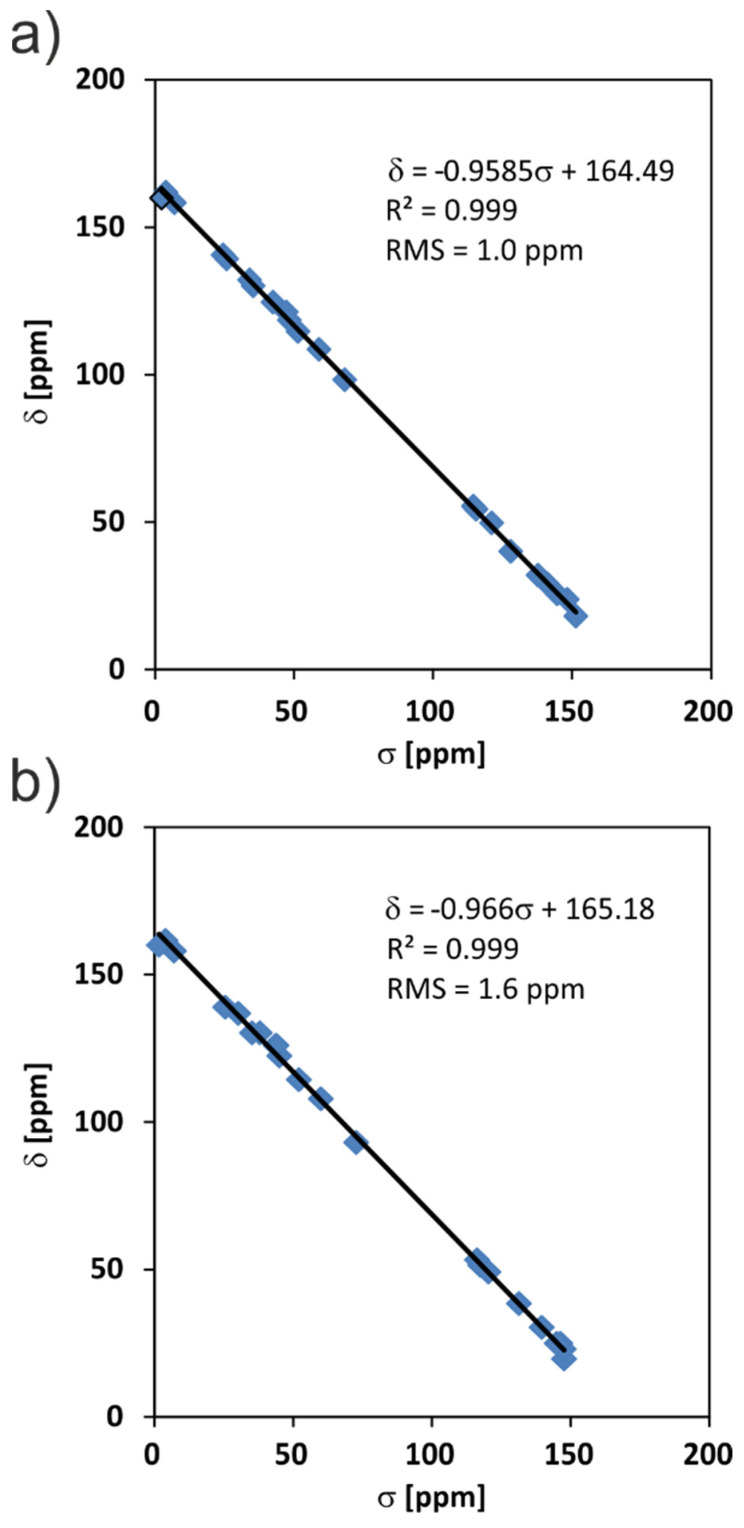
The correlation of experimental ^13^C chemical shift values (δ) and calculated nuclear shielding (σ) values of the **MBS** (**a**) and **MBS**^HCl^ (**b**).

The final assignments were performed with the help of advanced theoretical calculations using the gauge including projector augmented waves (GIPAW) methodology, which is currently the gold standard for computing NMR parameters in solid-state systems [34,40,41]. More technical details regarding the employed computational method are included in the Methods Section. The plots of correlation between experimental and theoretical NMR values are presented in Figure 9 and yield RMS values as low as 1.0 and 1.6 ppm for **MBS** and **MBS**^HCl^, respectively. Each graph contains over 19 unique experimental chemical shifts as linear functions of theoretical results, which finally confirms the high confidence of the presented assignment of signals.

**Figure 10:**
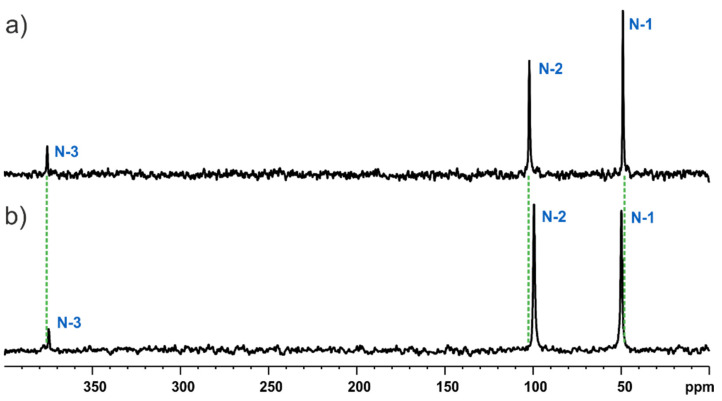
^15^N CP MAS NMR spectra of **MBS** recorded at a spinning rate of 12 kHz (**a**) and 25 kHz (**b**) at an ambient temperature. Asterisks indicated spinning sidebands.

Although both investigated models have significant structural differences, their ^15^N CP MAS spectra, or, more specifically, nitrogen signal positions, are nearly identical (Figure 10). This is a bit surprising especially for atom N-2 which is the protonation site undergoing reaction with HCl. The chemical properties of this position are significantly different in the protonated (**MBS**^HCl^) and non-protonated (**MBS**) forms but remain almost magnetically equivalent. This is sp^3^-hybridised type nitrogen for which, in the literature, a similar effect has been observed in the liquid state. This observation was explained by the fact that, although protonation of the nitrogen lone pair resulted in lowering the shielding effect, it was compensated for by increasing the shielding protonation effect which came from the N–C bonds, therefore a totally vanished effect [46]. Since our investigation was made for solid-state forms, there is an additional effect, as crystal structure packing may have an influence on NMR parameters. We employed the GIPAW methodology to estimate the influence of intermolecular interactions on the observed NMR chemical shift of nitrogen. For the purpose of this investigation, we created theoretical models of isolated **MBS** molecules in a box of size 27,000 Å^3^ to prevent any intermolecular interactions. Table S1 presents the comparison of theoretical ^15^N chemical shift values using these two conditions. It is easily seen that the maximum crystal packing effect on the ^15^N chemical shifts is up to 10 ppm and has similar impacts for the **MBS** as well as **MBS**^HCl^ form.

### 3.3. Fast MAS NMR Final Validation of Structures by Means of ^1^H Chemical Shifts

**Figure 11:**
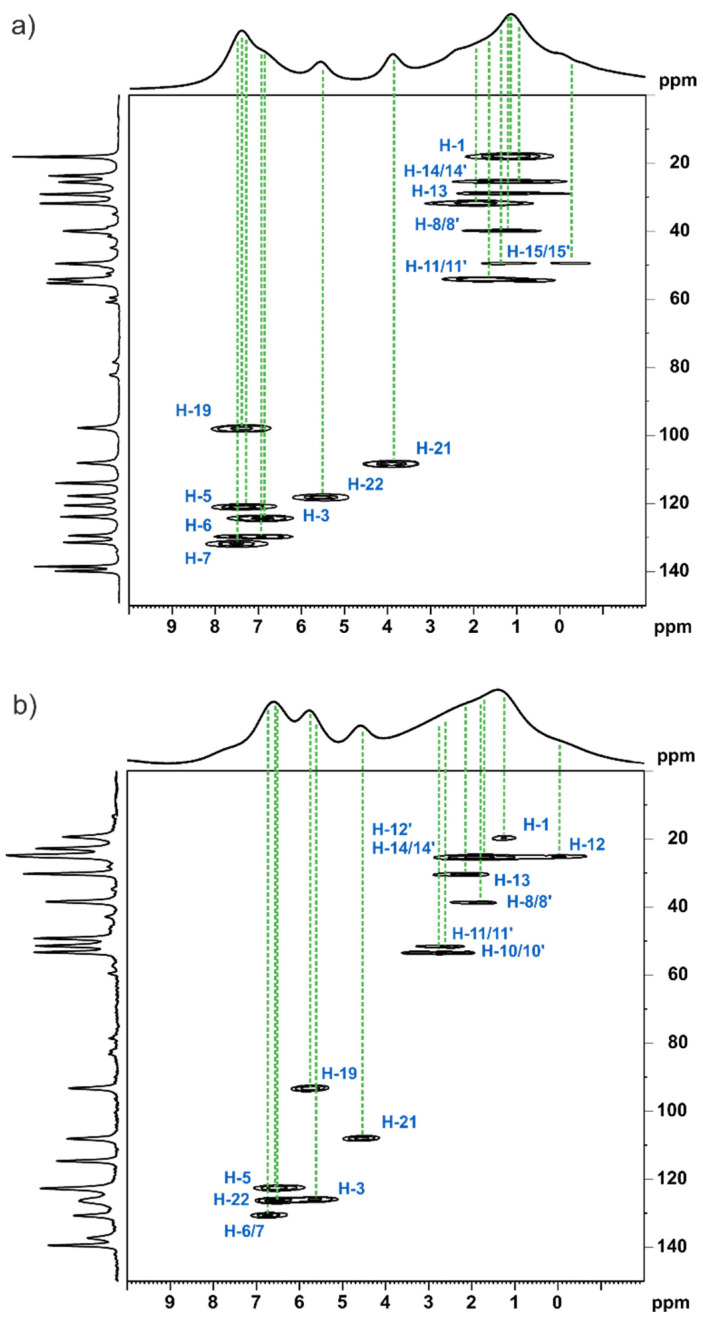
^13^C–^1^H invHETCOR MAS NMR spectra of **MBS** (**a**) and **MBS**^HCl^ (**b**) recorded at 90 °C with a spinning rate of 60 kHz at a ^1^H Larmor frequency of 600.1 MHz with a second ^13^C → ^1^H CP contact time of 100 μs. A one-pulse ^1^H MAS spectrum is shown at the top and ^13^C CP MAS in the left.

In this section, we describe the solid-state MAS NMR characterisation of both studied forms. We applied the fast MAS technique, which allowed us to record the ^1^H NMR spectra with reasonable resolution. The fast MAS opened a new area in the solid-state NMR enabling acquisition of 2D heteronuclear experiments with indirect inverse (inv) observations via ^1^H. In our study, we applied the ^13^C–^1^H invHETCOR MAS NMR experiment that allowed us to record ^1^H–^13^C correlations. Figure 11 shows the ^13^C–^1^H invHETCOR MAS spectra recorded with a spinning rate of ν_R_ = 60 kHz and a short second contact time. This method allowed us to observe correlations corresponding to short C^…^H distances only. In that way, it was possible to accurately assign most of the ^1^H directly bonded to the ^13^C.

**Figure 12:**
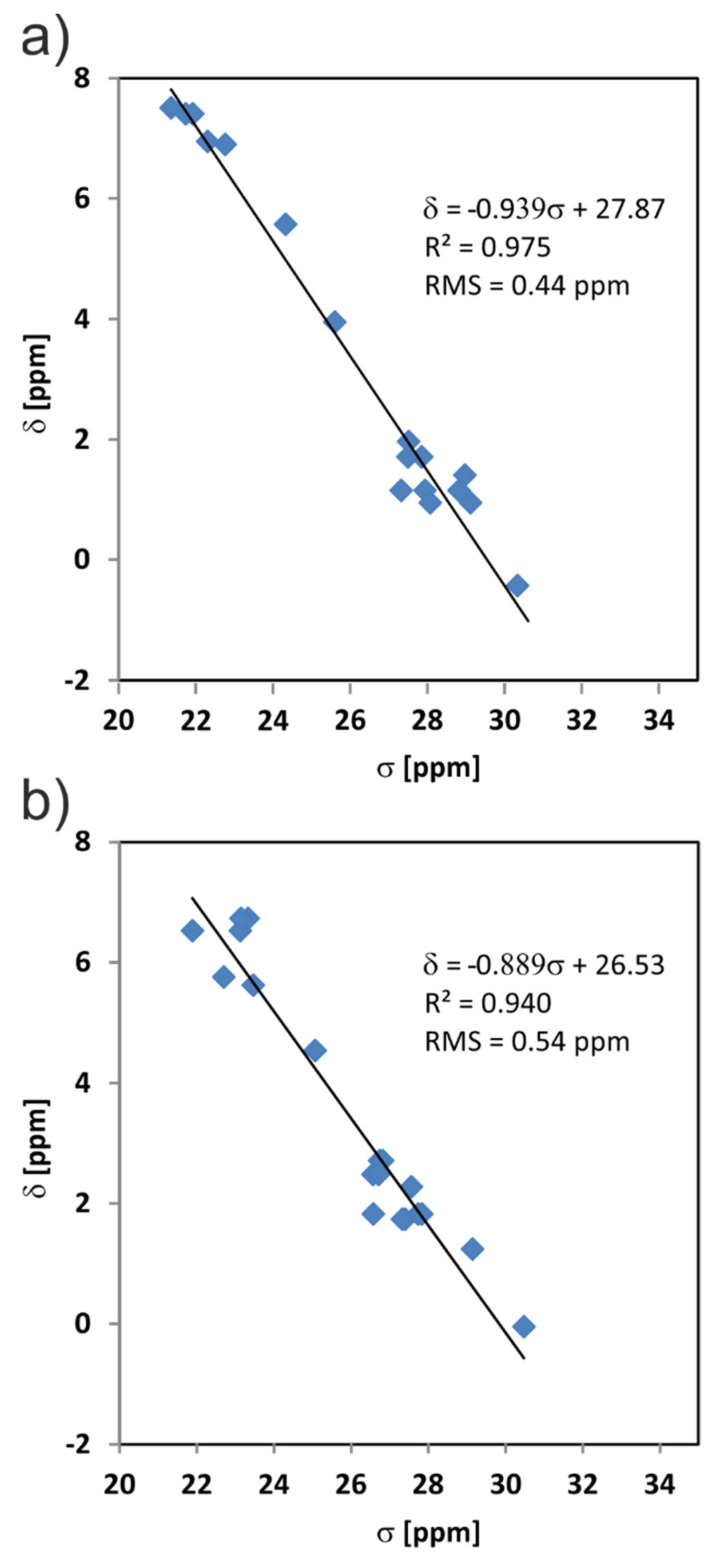
The correlation of experimental ^1^H chemical shift values (δ) and calculated nuclear shielding (σ) values of the **MBS** (**a**) and **MBS**^HCl^ (**b**).

One of the advantages of the ^1^H fast MAS spectra is the fact that the ^1^H nuclei are very sensitive probes for structural intermolecular factors. The well-established method to verify structure determined by X-ray diffraction is to compare the ^1^H experimental solid-state NMR chemical shifts with the GIPAW calculated results and then evaluate the quality of such correlation [47,48,49,50,51,52]. Figure 12 shows the correlation between experimental and theoretical results for the **MBS** and **MBS**^HCl^ crystal structures (after DFT geometry optimisation). Table S2 presents the numerical values. A high-quality agreement is observed between the experimental and GIPAW calculated chemical shifts. The root-mean-squared deviation (RMSE) values are up to 0.5 ppm. In the literature, we have found that RMSE values below 0.5 ppm relate to excellent agreement between experimental and calculated values [53]. Since our results are close to this limit, it proves that the single-crystal X-ray solutions of both **MBS** and **MBS**^HCl^ structures defined the bulk material.

## 4. Conclusions

The structural studies of chemical substances that have potential medical applications are now needed more than in the past. In this study, we reported the comprehensive solid-state characterisation of AND-1184 (**MBS**) and its hydrochloride form (**MBS**^HCl^) as potential agents for the treatment of dementia. The studied forms were both characterised by using various structural techniques. Since both forms crystalise in different crystal systems exhibiting different molecular conformations—namely, P2_1_/c and C2/c for **MBS** and **MBS**^HCl^, respectively, their ^13^C solid-state NMR spectra represent easily accessible fingerprints of studied materials. The advanced solid-state NMR experiments, combined with quantum-chemical calculations, allowed us to assign all the ^13^C and most ^1^H signals identifying the crucial differences between protonated and non-protonated forms. Finally, the single-crystal X-ray method and the PISEMA solid-state NMR experiment identified the **MBS** structure as a completely rigid system without significant dynamic processes. Even the large chemical changes from **MBS** to **MBS**^HCl^ do not change this feature significantly. The **MBS**^HCl^ exhibit only limited dynamic motion of the aromatic part of the molecule and have surprisingly similar ^15^N spectra to the non-protonated form. **MBS** and **MBS**^HCl^ differ significantly in their solubility, which has a strong influence on bioavailability, and thus on the therapeutic effect. In our study, we showed that the more soluble **MBS**^HCl^ (as compared with **MBS**) can be easily obtained using a mechanochemical approach. Such a procedure is quick, ecologically, and economically justified.

## Figures and Tables

**Table 1:** Experimental details.

Crystal Data	
Chemical formula	C_22_H_26_FN_3_O_3_S
M_r_	431.52
Crystal system, space group	Monoclinic, P2_1_/c
Temperature (K)	100
a, b, c (Å)	13.9487 (1), 8.4474 (1), 17.5121 (2)
β (°)	92.163 (1)
V (Å^3^)	2061.98 (4)
Z	4
Radiation type	Cu Kα
Diffractometer	XtaLAB Synergy, Dualflex, Pilatus 300 K
No. of measured, independent, and observed [I > 2σ(I)] reflections	59,889, 4409, 3970
R_int_	0.060
(sin θ/λ)_max_ (Å^−1^)	0.637
R[F^2^ > 2σ(F^2^)], wR(F^2^), S	0.035, 0.091, 1.04
No. of reflections	4409
No. of parameters	276
Δ〉_max_, Δ〉_min_ (e Å^−3^)	0.51, −0.48

**Table 2:** Hydrogen-bond geometry (Å, °) for 1.

D—H···A	D—H	H···A	D···A	D—H···A
N1—H1···N2 ^i^	0.87 (2)	2.13 (2)	2.9863 (18)	168.4 (19)
C5—H5···O1 ^ii^	0.93	2.49	3.3683 (18)	159
C19—H19···O2 ^iii^	0.93	2.59	3.2589 (17)	130

Symmetry codes: ^i^ −x + 1, y + 1/2, −z + 1/2; ^ii^ −x + 1, −y + 2, -z + 1; ^iii^ x + 1, y − 1, z.

## Data Availability

Data is contained within this article or supplementary material. CCDC: 2115171 contains the supplementary crystallographic data for this paper. The data is provided free of charge by The Cambridge Crystallographic Data Centre via www.ccdc.cam.ac.uk/structures.

## Supplementary Materials

The following are available online at https://www.mdpi.com/article/10.3390/ma14237175/s1. Figure S1: ^19^F MAS NMR spectra of **MBS** and **MBS**^HCl^ recorded at a spinning rate of 12 kHz at an ambient temperature, Figure S2: 2D PISEMA MAS spectra for sample **MBS** and **MBS**^HCl^. Spectra were acquired at a 13 kHz spinning rate, Table S1: ^15^N NMR GIPAW calculated chemical shifts for **MBS** and **MBS**^HCl^ in the box and crystal, Table S2: ^1^H NMR experimental and GIPAW calculated chemical shifts for **MBS** and **MBS**^HCl^.
