# Supplementary material for: Single-Crystal X-ray and Solid-State NMR Characterisation of AND-1184 and Its Hydrochloride Form
Source: Materials (Basel). 2021 Nov 25;14(23):7175. doi: 10.3390/ma14237175 (PMC8658117; doi:10.3390/ma14237175)
Supplement: Supplementary file 1 [file materials-14-07175-s001.zip › materials-1448058-supplementary.pdf]

materials

Supplementary Materials

Single-Crystal X-ray and Solid-State NMR Characterisation of AND-1184 and Its Hydrochloride Form

Tomasz Pawlak [1,*], Małgorzata Szczesio [2], and Marek J. Potrzebowski [1]

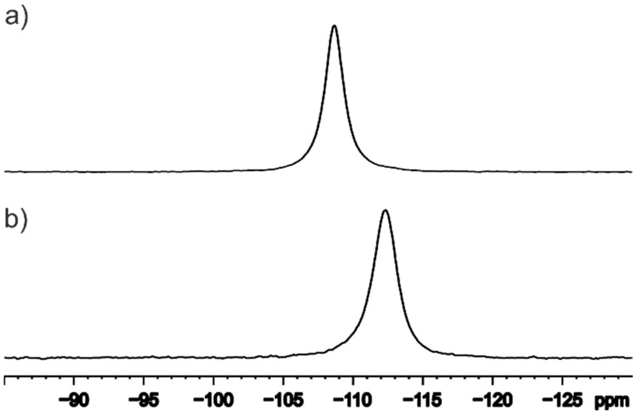

Figure S1. ^{19}F MAS NMR spectra of **MBS** (**a**) and **MBS**^{HCl} (**b**) recorded at a spinning rate of 12 kHz at the ambient temperature.

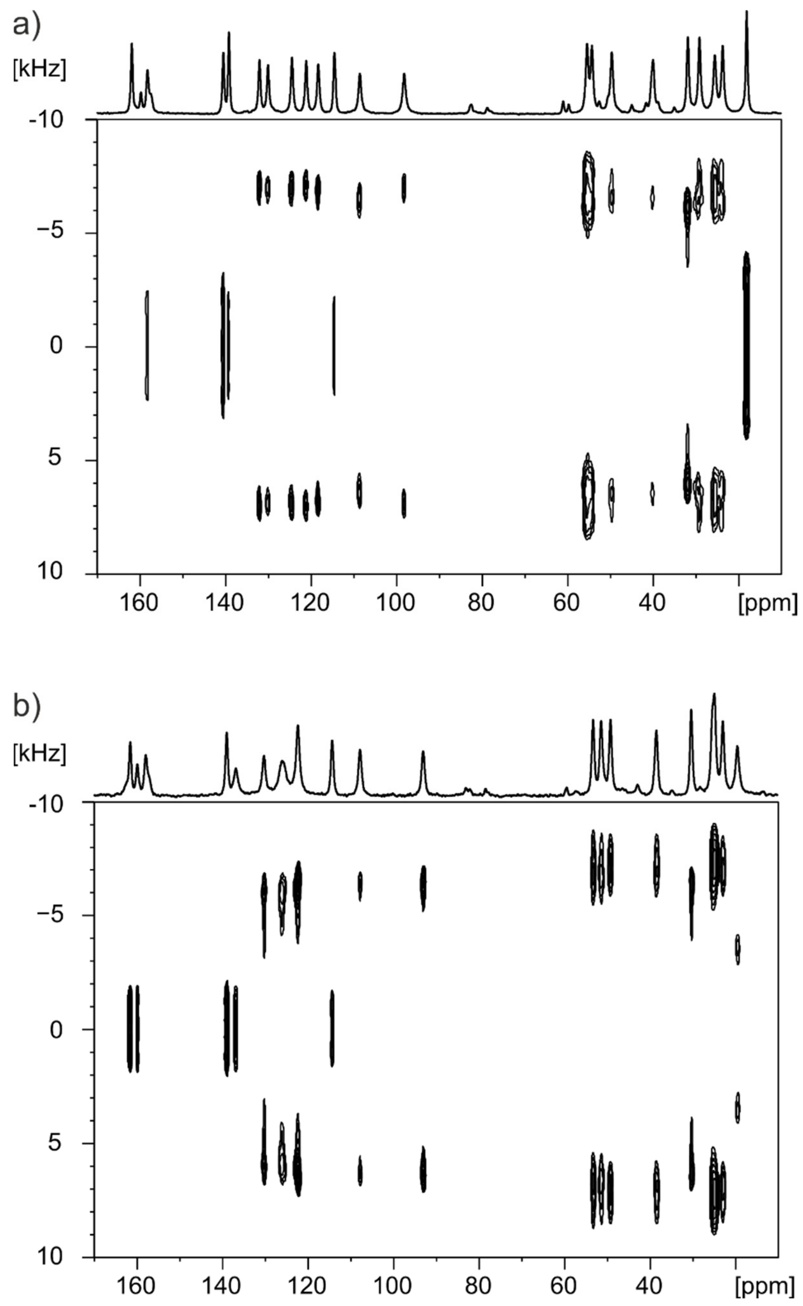

Figure S2. 2D ^{1}H-^{13}C PISEMA MAS spectra for sample **MBS** (**a**) and **MBS**^{HCl} (**b**). Spectra were acquired at a 13 kHz spinning rate.

Comment to the Figure S2:

We used 2D PISEMA MAS experiments to measure ^{13}C–^{1}H dipolar couplings and probe dynamic processes on the microsecond time scale. The splitting between the singularities in the F1 dimension of 2D PISEMA MAS spectra reflects the dipolar coupling between the specific carbon and closely located protons. The rigid limit for PISEMA is 23.3 kHz. The experimentally measured splitting values are smaller than the calculated coupling values because the observed splitting is reduced by a scaling factor. For the PISEMA MAS NMR experiment, the exact Hartmann–Hahn matching condition yields a scaling factor of 0.577 (cos 54.7°), and the expected splitting value is ca. 13.4 kHz (23.3 kHz × 0.577). Motional processes can be quantitatively probed by measuring the reduction in the dipolar coupling in comparison to the rigid limit. Figure S2 presents 2D ^{1}H–^{13}C PISEMA

MAS NMR spectra for **MBS** and **MBS**^{HCl}. Since all the protonated carbons have splitting around 13 kHz for **MBS** we shroud assign the absent of molecular motion to this system. However in the case of aromatic signals of **MBS**^{HCl} we observes slightly lower splitting values of around 11-12 kHz. Such observation can be interpreted as a low-amplitude wobbling of this part of molecule. In our study we did not observe reduction of splitting value below 8 kHz which corresponds to the high amplitude molecular dynamic.

Table S1. ^{15}N NMR GIPAW calculated chemical shifts (δ) [in ppm] (recalculated from GIPAW nuclear shieldings from equation $\delta = 218.7 - \sigma^{GIPAW}$) for **MBS** and **MBS**^{HCl} in the box and crystal.

Structure (position)	δ^{MBS} (crystal)	δ^{MBS} (in the box)	$\delta^{MBS\ HCl}$ (crystal)	$\delta^{MBS\ HCl}$ (in the box)
N-1	109.2	106.3	104.8	101.7
N-2	47.7	45.4	51.1	49.9
N-3	386.1	393.9	384.2	388.5

Table S2. ^{1}H NMR experimental and GIPAW calculated chemical shifts (δ) for **MBS** and **MBS**^{HCl}. GIPAW chemical shifts were recalculated from nuclear shielding according to the equations in Figure 12.

Structure (position)	δ^{MBS} Exp	δ^{MBS} GIPAW	$\delta^{MBS\ HCl}$ Exp	$\delta^{MBS\ HCl}$ GIPAW
H-1	1.15	0.69	1.23	0.38
H-3	6.89	6.47	5.62	5.80
H-5	7.40	7.45	6.52	6.12
H-6	6.94	6.90	6.73	5.93
H-7	7.50	7.81	6.73	6.11
H-8	1.15	2.12	1.73	2.05
H-8′	1.15	1.53	1.73	2.11
H-9	not visible	-1.85	not visible	0.57
H-9′	not visible	-0.61	not visible	2.74
H-10	not visible	1.27	2.70	2.60
H-10′	not visible	1.49	2.70	2.68
H-11	1.71	1.62	2.48	2.85
H-11′	1.71	1.95	2.48	2.71
H-12	not visible	0.28	1.82	1.73
H-12′	not visible	1.05	-0.08	-0.88
H-13	1.96	1.93	2.27	1.89
H-14	0.94	0.41	1.82	2.84
H-14	0.94	1.40	1.82	1.64
H-15	-0.42	-0.75	not visible	2.00
H-15′	1.40	0.55	not visible	3.53
H-19	7.40	7.27	5.75	6.53
H-21	3.94	3.76	4.53	4.27
H-22	5.57	4.98	6.52	7.30
